# Inflammatory angiomyolipoma of the liver: a rare hepatic tumor

**DOI:** 10.1186/1746-1596-7-122

**Published:** 2012-09-15

**Authors:** Yang Liu, Jian Wang, Xu-Yong Lin, Hong-Tao Xu, Xue-shan Qiu, En-Hua Wang

**Affiliations:** 1 Department of Pathology, the First Affiliated Hospital and College of Basic Medical Sciences, China Medical University, Shenyang, 110001, China; 2 Institute of Pathology and Pathophysiology, China Medical University, Shenyang, 110001, China

**Keywords:** Angiomyolipoma, Perivascular epithelioid cell, Inflammatory, Liver

## Abstract

Angiomyolipoma (AML) is a rare mesenchymal neoplasm of the tumor, composed of a varying heterogeneous mixture of three tissue components: blood vessels, smooth muscle and adipose cells. Hepatic AML may demonstrate a marked histological diversity. We herein present one case of hepatic AML exhibiting prominent inflammatory cells in the background, which happened in a 61-year-old Chinese female patient, without signs of tuberous sclerosis. Histologically, the striking feature was the infiltration of numerous inflammatory cells in the background, including small lymphocytes, plasma cells, and eosnophils. The tumor cells were spindled and histiocytoid in shape, with slightly eosinophilic cytoplasm, and arranged along the vessels or scattered among the inflammatory background. Sinusoid structure was obviously seen in the tumor. Mature adipocytes and thick-walled blood vessels were focally observed at the boundaries between the tumor and surrounding liver tissues. The tumor cells were positive immunostaining for HMB-45, Melan-A, and smooth muscle actin. The inflammatory AML should be distinguished from other tumors with inflammatory background such as inflammatory myofibroblastic tumor and follicular dendritic cell tumor and deserves wider recognition for its occurrence as a primary hepatic tumor.

**Virtual slides:**

The virtual slide(s) for this article can be found here: http://www.diagnosticpathology.diagnomx.eu/vs/1828633072762370

## Background

Hepatic angiomyolipoma is a rare, benign, hepatic mesenchymal neoplasm found in both males and females, and most commonly in adult females. Angiomyolipoma occurs most commonly in the kidneys. The liver represents the second most frequent site of involvement [1]. Histologically similar to those in the kidney, hepatic AML consists of a mixture of myoid cells, adipose tissue and thick-walled vessels. They may have variable morphologic features and are positive for HMB-45, but negative for hepatocyte paraffin-1 (Hepar-1) and S100 protein [1,2]. According to the line of differentiation and predominance of tissue components, the tumors were subcategorized into mixed, lipomatous (> = 70% fat), myomatous (<=10% fat), and angiomatous type. The most common type is the mixed type which comprises sheets of epithelioid muscle cells admixed with islands of adipocytes and abnormal vessels. The lipomatous and myomatous patterns were regarded as morphologic variations on a continuous spectrum, depending on the degree of adipose and myoid differentiation. Myomatous type was more common in the liver than in the kidney [3]. Angiomatous AML contained many large thick-walled vessels and radiologically may be misinterpreted as intrahepatic arterial aneurysm. According to the predominant component, growth pattern, cell type, and other features, the tumors were subcategorized into trabecular, pelioid and inflammatory variants. Of these, inflammatory or pelioid pattern usually presents as a focal finding within the tumor, but very rarely, they become the predominant pattern [4], creating great diagnostic confusion with other tumors such as inflammatory myofibroblastic tumor (IMT), follicular dendritc cell (FDC) tumor and other hepatic mesenchymal neoplasms. The authors herein present such a case of hepatic AML with a predominantly inflammatory pattern, also known as inflammatory AML.

## Case presentation

### Clinical history

A 63-year-old woman was admitted to the First Affiliated Hospital of China Medical University in June of 2010 for further examination of the liver tumor which was detected by ultrasonography in the annual health check. Physical examination showed no abnormalities. Hematological and chemical studies, including tumor markers such as α-fetoprotein and carcinoembrionic antigen, gave normal results. Hepatitis virus markers, such as hepatitis B surface antibody, hepatitis B surface antigen and hepatitis C antibody, were all negative. Conventional ultrasonography revealed well-demarcated isoechoic tumor with a diameter of 30 mm in the segment V of the liver. The spleen, pancreas, and kidneys were without any focal lesions. There are no pathognomonic clinical signs for tuberous sclerosis. The patient did not consent to tumor biopsy, and we could not rule out the possibility of malignancy due to the result of ultrasonography and CT scan. The patient desired to undergo tumor resection on her own initiative, and partial hepatectomy was performed. The patient was alive with no tumor recurrence or metastasis at 2 years of follow-up.

### Gross features

Gross examination showed an elastic hard mass with a diameter of 30 mm. The tumor did not have a capsule, but it was clearly demarcated from the normal hepatic parenchyma. The tumor was grayish-white on cut surface.

### Microscopic features

**Figure 1:**
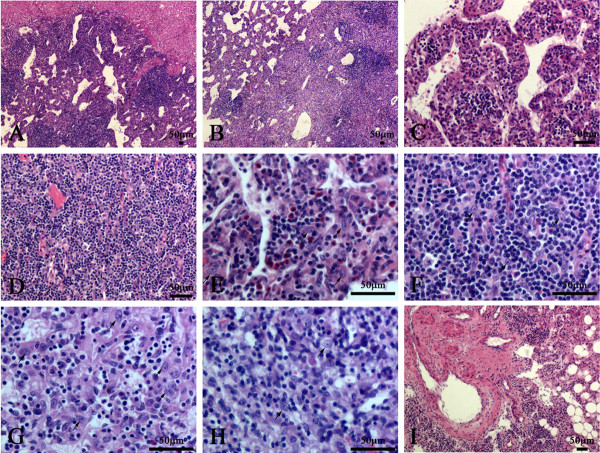
**Histological features of this case.A**: The neoplasm was demarcated from the surrounding liver tissues with relative clear boundary. **B**: The neoplasm showed a solid cellular growth pattern and dilated vascular channels. **C**: The cavernous-like vascular areas were composed of dilated vascular channels lined by monolayer flat endothelial cells, and separated by cellular septa. **D**: The solid cellular areas contained many capillaries with the narrow or collapsed lumen. **E** and **F**: The tumor was characterized by the infiltration of numerous inflammatory cells in the background, including small lymphocytes, plasma cells, and eosnophils. **G** and **H**: The cellular areas and the septa of cavernous-like vascular areas were composed of spindled and histiocytoid cells (arrow) with slightly eosinophilic cytoplasm and small central nucleoli. **I**: Mature adipocytes and thick-walled blood vessels were focally observed at the boundaries between the tumor and surrounding liver tissues.

The neoplasm was demarcated from the surrounding liver tissues with relative clear boundary, presenting with a solid cellular growth pattern and abundant vascularity with frequently dilated vascular channels (Figure. 1A–D). The tumor was characterized by the infiltration of numerous inflammatory cells in the background, including small lymphocytes, plasma cells, and eosnophils (Figure. 1C–F). The proportion of tumor area with inflammatory infiltration was more than 80%. The tumor cells were spindled and histiocytoid in shape, with slightly eosinophilic cytoplasm and small central nucleoli, and arranged along the vessels or scattered among the inflammatory background (Figure 1E–H). Pleomorphism is absent and mitotic figures are barely seen. Mature adipocytes and thick-walled blood vessels were focally observed at the boundaries between the tumor and surrounding liver tissues. The mature adipocyte component was less than 5% of the whole tumor and interrupted by sheets of histiocytoid and spindle myoid cells (Figure 1I). No necrosis, hemorrhage, or cyst formation was observed in the tumor. No sclerosing cholangitis was observed in the intrahepatic bile ducts of the surrounding liver tissues.

### Immunohistochemistry

**Figure 2:**
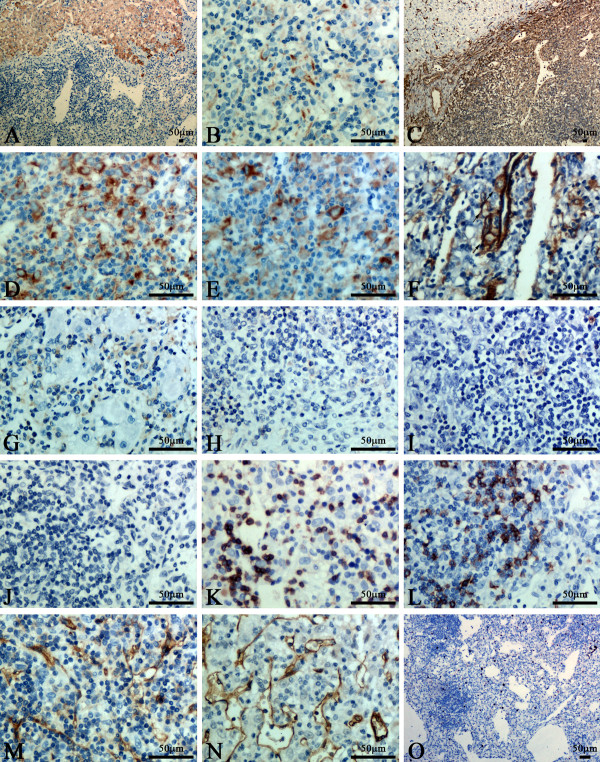
**Immunohistochemical staining.A**: The liver tissues surrounding the tumor were diffusely positive staining for AE1/AE3. **B**: The histiocytoid cells were faintly positive staining for AE1/AE3. **C**-**E**: The histiocytoid cells were strongly diffuse positive staining for vimentin, HMB-45, melan-A. **F**: The histiocytoid cells were focally positive staining for smooth muscle actin, especially the cells around blood vessel. **G**: Scattered tumor cells were positive for CD68. **H**-**J**: The histiocytoid cells were negative staining for CD21, S100 and ALK. **K** and **L**: The lymphocytes among the histiocytoid cells were mainly positive for CD3 and focally positive for CD20, whereas the histiocytoid cells were negative. **M** and **N**: CD31 and CD34 underlined the rich vascular channels, whereas the histiocytoid cells were negative. **O**: Ki67 index was about 5%.

The immunohistochemical study showed that the histiocytoid cells were faintly positive for AE1/AE3 (Figure 2A and B), strongly diffuse positive for vimentin (Figure 2C), HMB-45 (Figure 2D), Melan-A (Figure 2E), focally positive for smooth muscle actin (SMA) (Figure 2F), and occassionally positive for CD68 (Figure 2G). They were strictly negative for CD21 (Figure 2H), S100 (Figure 2I), ALK (Figure 2J) CD1α, Hepar-1, CD35, CD10, CD23, CD117, DOG-1, synaptophysin and chromogranin A (data not shown). The lymphocytes among the epithelioid cells were mainly positive for CD3 (Figure 2K) and focally positive for CD20 (Figure 2L). Finally, CD31 and CD34 underlined the rich vascular channels (Figure 2M and N). Ki67 index was about 5% (Figure 2O).The results were listed in Table 1.

**Table 1:** Panel of Immunohistochemical Stains

**Immunohistochemical Stain**	**Result**
Pan-cytokeratin (AE1/AE3)	+, faintly
vimentin	+
HMB-45 (melanoma-associated marker)	+
melan-A	+
smooth muscle actin (SMA)	+, focally
CD68	+, occassionally
hepatocyte paraffin-1 (Hepar-1)	-
S100 protein	-
ALK	-
CD1α	-
CD21	-
CD35	-
CD3	-
CD20	-
CD23	-
CD10	-
CD31	-
CD34	-
CD117	-
DOG-1	-
synaptophysin	-
chromogranin A	-
Ki67	about 5%

## Discussion

Hepatic angiomyolipoma, a member of the family of tumors showing differentiation resembling perivascular epithelioid cells, was first described by Ishak in 1976 [2]. Regardless of their location, the tumors in this family share mature fat, thick-walled poorly organized blood vessels and spindle-epithelioid myoid cells. Hepatic AML is a rare mesenchymal tumor of the liver. Tsui et al. [2] described many morphologic variations of hepatic AML which reflect the variable lineage and degree of differentiation of the myoid cells. The histologic patterns described in the literature include lipomatous, myomatous, angiomatous, trabecular, pelioid, inflammatory and mixed pattern [2,5]. Trabecular variant of AML was characterized by a rich vascular framework, and the tumor cells were arranged in clusters and surrounded by dilated sinusoidal vessels [6,7]. Other unusual architectural patterns such as pelioid and inflammatory ones were usually present as focal finding, but sometimes they may exist as a pure pattern [4,8,9] which makes it difficult to distinguish with other hepatic tumors.

Although hepatic AML has various types or variants and mimics various hepatic neoplasms, it can still be recognized or suspected on morphologic grounds. The clues to the diagnosis are the 3 characteristic components (blood vessels, smooth muscle, and fat tissue) and diagnostic myoid component which may exist in epithelioid, spindle, and intermediate forms. It has been speculated that the distinctive epithelioid cells are primitive mesenchymal cells with an ability to differentiate toward both myoid and adipose cells. Immunohisochemically, these cells are strongly positive for HMB-45 and smooth SMA.

In this case, the striking feature was the infiltration of numerous inflammatory cells with scattered histiocytoid cells among them, so the first diagnosis come to our mind is FDC tumor instead of inflammatory AML. It’s also hard to totally rule out inflammatory pseudotumor (IPT) and IMT on morphologic grounds, so we perform immunostaining to distinguish between them. To our surprise, the immunophenotype (CD21-, CD35, S100-, SMA focal +, ALK-) overthrows the diagnosis of FDC tumor, IPT and IMT, so we reviewed this case carefully and found some scattered adipocytes and thick-walled blood vessels at the boundaries between the tumor and surrounding liver tissues (As shown in Figure. 1I). This indicates the diagnosis of AML, so HMB-45 and Melan-A were added to stain and the result (HMB-45+, Melan-A+) demonstrated the diagnosis of AML. We searched the similar case on PubMed (http://www.ncbi.nlm.nih.gov) and found the inflammatory variant of AML may share the similar feature with our case, so the final diagnosis is hepatic inflammatory AML.

Inflammatory AML should be distinguished from other primary or metastatic hepatic tumors especially those with a prominent inflammatory cell infiltration in the background, such as IPT, IMT, FDC tumor, lipomatous tumors, sarcomatoid carcinoma with prominent lymphocytic infiltration, poorly differentiated hepatocellular cell carcinoma, gastrointestinal stromal tumors and metastatic renal cell carcinoma [10-16]. The so-called IPT and IMT are the first differential diagnosis which should be distinguished from Inflammatory AML because of the heavy inflammatory infiltration in the background. The so-called IPT is composed of inflammatory cells and some reactive fibroblasts or collagen-rich connective tissue [13]. While, IMT is thought to be neoplastic and harbor a clonal cytogenetic aberration that activates the ALK-receptor tyrosine kinase gene at 2p23. IMT consists of spindled myofibroblasts which are positive for SMA and ALK [10,12,14]. The adipose tissue and sinusoidal vessels are usually absent in IPT or IMT. The myofibroblastic cells in IMT are predominantly spindled, and epithelioid myofibroblastic cells are absent or only very few if present. In addition, immunostaining will be helpful to distinguish between them because IPT and IMT are negative for HMB-45. Another important differential diagnosis of inflammatory AML is FDC tumor, which is not common in the liver and usually shows a heavy lymphocytic infiltration in the background. This tumor can have occasionally inflammatory pseudo-tumor-like variant which occurs exclusively as primary tumor in the liver and spleen. However, the nuclei of FDC tumor usually show vesicular chromatin and distinct nucleoli. The FDC tumor does not have prominent dilated sinusoidal and thick-walled blood vessels, and the tumor cells are negative for HMB-45 but positive for CD21 and CD35 [11,15,16].

Primary or metastatic lipomatous tumor of the liver is extremely rare and may occasionally show an inflammatory background [17], but thick-walled blood vessels and the perivascular arrangement of epithelioid cells are seldom seen in these tumors. In addition, melanin marker (HMB-45) and muscle marker (SMA) will be helpful to diagnosis [17]. Sarcomatoid carcinoma always demonstrates obvious cytological atypia and does not have the thick-walled vessels and adipose tissue. In difficult cases, stains for CK and EMA as well as HMB-45 should be able to distinguish this tumor from inflammatory AML. Occasionally, inflammatory AML might be mistaken for hepatocellular carcinoma when a trabecular pattern is focally present or the epithelioid cells show clear cytoplasm [7,18]. However, hepatocellular carcinoma mostly occurs in a background of cirrhosis and usually lacks mature adipose tissue. Hepar-1, HMB-45 and SMA will be helpful to distinguish between them. In addition, other spindled cell tumors such as epithelioid leiomyosarcoma and inflammatory malignant fibrous histiocytoma may occasionally occur in the liver with an inflammatory background and histologically mimic inflammatory AML. However, the prominent nuclear atypia, frequent mitotic figures, and negativity for HMB-45 would be helpful to diagnosis. Metastatic gastrointestinal stromal tumors may show cytoplasmic clearing but typically do not have the adipose tissue and inflammatory background. The sinusoidal vascular structure is also absent in gastrointestinal stromal tumor. Finally, poorly differentiated cholangiocarcinoma or other metastatic carcinoma such as renal cell carcinoma may have a prominent inflammatory background occasionally. In the differential diagnosis with these tumors, a panel of antibodies including HMB-45, Hepar-1, AFP, CK18, and CK19 would be helpful for the correct diagnosis.

The treatment of hepatic AML is hepatectomy for large tumors and conservative follow-up for small ones. Most hepatic AMLs behave in a benign fashion, although malignant hepatic AML has been reported in the literature [19,20]. This phenomenon may attribute to the malignant transformation which has been reported in many tumors with different histological types [21-23]. Based on the criteria described by Nguyen [19], the differences between benign and malignant hepatic AML were summarized in Table 2. In this case, coagulative necrosis was not found, and the tumor was 30 mm in diameter. Moreover, the patient was alive with no tumor recurrence or metastasis at 2 years of follow-up. All these features support the diagnosis of benign hepatic AML. Since CD117 was negative in this case, careful follow-up of patients is recommended in this case. Inflammatory AMLs do not show any difference in prognosis from the classical AMLs. This variant of AML should be recognized and avoid misdiagnosing as other malignant or intermediate tumors such as hepatic FDC tumor and IMT, which require an active treatment regimen.

**Table 2:** Distinguishing features of benign and malignant hepatic AML

	**Benign HAML**	**Malignant HAML**
Coagulative necrosis	No	Yes
Tumor size	8 ± 7 cm	>10 cm
Evidence of metastasis or death attributed to the tumor	No	Yes
Expression of CD117	Yes	No

HAML: hepatic AML.

## Conclusion

In this case, the tumor was nearly mistaken for IMT or FDC tumor which indicates it’s hard to distinguish between them in the practical work. The 3 characteristic components (myoid cells, adipose tissue and thick-walled vessels) maybe indicates a diagnosis of hemangioblastoma, but don’t exclude the probability when one or two components were hardly seen, especially in the pelioid and inflammatory variant of AML. Therefore, AML must be included in the differential diagnosis of hepatic tumors with histiocytoid appearance and inflammatory background to not underestimate this tumor in this location and so to better evaluate its real frequency and not establish wrongly a diagnosis of malignancy to this benign tumor. Using combination of immunohistochemistry may be helpful to some rare hepatic tumors.

## Consent

Written informed consent was obtained from the patient for publication of this case report and accompanying images. A copy of the written consent is available for review by the Editor-in Chief of this Journal.

## Competing interests

The authors declare that they have no competing interests.

## Authors’ contributions

YL analyzed the data and wrote the manuscript as a major contributor. JW, XL and HX helped to perform the immunochemical staining. XQ and EW helped to revise the discussion section of this manuscript. All authors have read and approved the final manuscript.
